# Establishment of Structure-Function Relationship of Tissue Inhibitor of Metalloproteinase-1 for Its Interaction with CD63: Implication for Cancer Therapy

**DOI:** 10.1038/s41598-020-58964-x

**Published:** 2020-02-07

**Authors:** Richard B. Warner, Abdo J. Najy, Young Suk Jung, Rafael Fridman, Seongho Kim, Hyeong-Reh Choi Kim

**Affiliations:** 1 Department of Pathology, Wayne State University School of Medicine, Detroit, MI 48201 USA; 2 Department of Oncology, Barbara Ann Karmanos Cancer Institute, Wayne State University School of Medicine, Detroit, MI 48201 USA; 3 Present Address: Department of Chemistry, Southern Utah University, Cedar City, UT 84720 USA; 4 Present Address: Pusan National University College of Pharmacy, Busan, 46241 South Korea

**Keywords:** Breast cancer, Cell signalling

## Abstract

Tissue inhibitor of metalloproteinases-1 (TIMP-1) is a pleiotropic protein, promoting both tumor-suppressive and tumor-promoting activities. While TIMP-1 is primarily known as an endogenous inhibitor of matrix metalloproteinases (MMPs) and thus associated with tumor cell invasion, clinical studies demonstrated increased expression of TIMP-1 and its association with poor prognosis in cancer. Non-MMP-inhibitory and oncogenic functions of TIMP-1 are mediated by induction of intracellular signaling via its cell surface receptor CD63, a tetraspanin. The present study investigates the structure-function relationship of TIMP-1 for its interaction with CD63, which may eventually help design a novel approach for targeting TIMP-1’s pro-oncogenic activity without interfering its tumor suppressive MMP-inhibitory function. Importantly, our analysis includes TIMP-1/CD63 interactions at the cell surface of live cells. Here, we demonstrate that the 9 C-terminal amino acid residues of TIMP-1 and the large extracellular loop of CD63 are required for their interaction. Considering that the N-terminal half of TIMP-1 is sufficient for TIMP-1’s MMP-inhibitory activity, we propose that those C-terminal amino acid residues are a potentially targetable motif of TIMP-1 oncogenic activity. As a proof of concept, we present the potential for the development of neutralizing antibodies against the C-terminal motif of TIMP-1 for disruption of TIMP-1 interaction with CD63 and the subsequent signal transduction.

## Introduction

Tissue inhibitor of metalloproteinases-1 (TIMP-1) is a founding member of the TIMP family that comprises four members, TIMP-1 to TIMP-4, which as a whole act as major inhibitors of metalloproteinases including the matrix metalloproteinases (MMPs) and members of a disintegrin and metalloproteinase domain (ADAM) family of proteases^1^. Although this is an important tumor-suppressive function of TIMP-1, accumulating evidence has shown that TIMP-1 can elicit tumor-promoting effects via cell signaling independent of its MMP inhibitory activity^2–6^. The ability of TIMP-1 to regulate cell proliferation and survival was first reported when TIMP-1 was originally identified as a humoral factor that enhanced the growth of human erythroid progenitor cells^7,8^. Later studies established the ability of TIMP-1 to support cell survival in a variety of cells including carcinoma, lymphoma, immune cells, and endothelial cells^5,9^. Importantly, clinical studies clearly demonstrated the association of TIMP-1 expression with therapy resistance and poor prognoses in many types of cancers [^10–13^ and references therein], emphasizing the potential significance of TIMP-1 as an oncogenic signaling molecule in human cancers. Our discovery of CD63 as a cell surface receptor for TIMP-1 was one of the breakthrough findings to uncover the molecular actions of TIMP-1 as a signaling molecule for activation of cellular responses including cell survival and epithelial-to-mesenchymal transition (EMT)^2,3,6,14^. Previously, we demonstrated that TIMP-1 interactions with CD63 and subsequent activation of intracellular signaling programs do not require its MMP inhibitory domain^2,3,15^, indicating that TIMP-1’s opposite effects on tumor progression are mediated by two distinct functional domains. The goal of this study is to identify the CD63 binding motif of TIMP-1 that could be targeted to inhibit TIMP-1-mediated oncogenic signal transduction while preserving its tumor suppressive MMP-inhibitory functions. Here, we report that the 9 C-terminal amino acid residues of TIMP-1 are critical for its interactions with the cell surface receptor CD63. We also found that the large extracellular loop of CD63 is essential for TIMP-1 binding whereas the small extracellular loop of CD63 appears largely irrelevant. Utilizing the protein complementation assay (PCA), we confirmed that TIMP-1 interaction with CD63 occurs at the cell surface in live cells. In addition, we present evidence that the C-terminal motif is targetable, resulting in interference of TIMP-1 interactions with CD63 at the cell surface.

## Materials and Methods

### Antibodies

Antibodies were purchased as follows; anti-TIMP-1 Ab-2 (102 D1) monoclonal antibody (mAb) from Thermo Scientific (Fremont, CA), anti-TIMP-1 (EP1549RY) rabbit mAb and anti-CD63 mouse mAb from Millipore (Billerica, MA), anti-β-actin mAb and anti-mouse and rabbit IgG peroxidase conjugates from Sigma (St. Louis, MO), anti-transferrin receptor mAb from BD Transduction Laboratories (San Jose, CA), anti-GAPDH mAb from Santa Cruz Biotechnology, Inc. (Santa Cruz, CA), total and phospho T202/Y204 specific anti-p42/44 ERKs Abs from Cell Signaling (Danvers, MA), anti-Gaussia Luciferase pAb from Nanolight Technology (Pinetop, AZ).

### Primers and mutagenesis

All mutations or deletions were made by site-directed mutagenesis using QuikChange Mutagenesis II Kit (Agilent Technologies; Santa Clara, CA) as per manufacturer’s instructions. For the list of primers used see Supplemental Table 1.

### Protein complementation assay

Modified pEYFP-N1 and pECFP-C1 vectors (Clontech), in which the fluorescent protein genes were replaced by humanized Gaussia Luciferase N-terminal (GLucN) and C-terminal (GLucC) fragments, were obtained from Dr. James Granneman at our institute. The HNF4 vectors were a kind gift of Dr. Todd Leff at our institute. TIMP-1 and CD63 were cloned into these vectors in place of HNF4 (for primers used to make TIMP-1 and CD63 vectors see Supplemental Table 1). For all cases, the GLuc fragments were fused to the protein of interest via a flexible linker consisting of a 10 amino acid sequence (GlyGlyGlyGlySer GlyGlyGlyGlySer) as previously optimized for luciferase-fragment complementation assay^16^.

GLucN and GLucC fusion plasmids were co-transfected in a 1:1 ratio (400 ng DNA total/well) into HEK293FT cells in 24-well plates using Lipofectamine 2000 (Invitrogen) according to manufacturer’s instructions. Transfected cells were given fresh media after 5 hrs and cultured for an additional 17–19 hrs to allow expression of fusion proteins. Medium was exchanged with 220 ul/well of phenol-red free DMEM (Invitrogen) containing protease inhibitor cocktail (Roche; Indianapolis, IN). Cell membranes were disrupted by two cycles of freezing and thawing at −80 °C and room temperature. For each sample, 100 ul was transferred to a white 96-well plate (Thermo Scientific) for luminescence measurements. Next, coelenterazine (a natural substrate for luciferase, purchased from Nanolight Technology) was injected to a final concentration of 10 uM. Signal intensities (integrated over 10 seconds after 2 seconds injection delay) were read on a MicroLumat 96 LB + plate reader (Berthold Technologies; Oak Ridge, TN), or a Glomax 96 microplate luminometer (Promega; Madison, WI).

To perform PCA in live cells, HEK293FT cells were transfected as described above in 96-well clear-bottom white plates (Thermo Scientific, 200 ng DNA total/well). Without rupturing membranes by freeze-thaw cycles, coelenterazine was added into the live cell culture (100 ul/well of phenol-red free DMEM containing protease inhibitor cocktail) and signal intensities were measured as described above with white backing tape (Perkin Elmer; Boston, MA) applied to the bottom of the plate.

### Statistical analysis

Significant differences in averages of measured enzyme activity were assessed by unpaired two-sided t-tests after data were log-transformed to meet normality assumptions. P-values were adjusted for multiple comparisons using the Dunnett’s method if the comparisons were made to single control group and the Holm’s method if otherwise. P-values of < 0.05 were considered statistically significant.

## Results and Discussion

**Figure 1:**
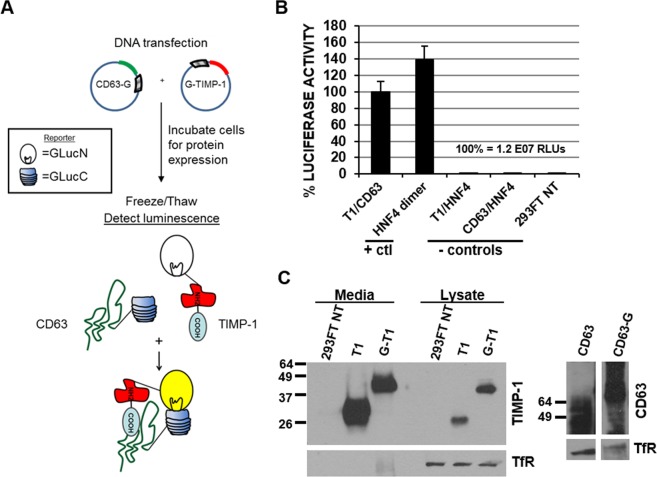
Protein complementation assay (PCA) for the analysis of TIMP-1 interactions with CD63. (**A**) Schematic diagram of luciferase activity restoration by recombined Gaussia luciferase fragments through interactions between TIMP-1 and CD63. (**B**) Luciferase activity was measured as a read-out for interactions between the proteins of interest using cell lysates prepared from HEK293FT cells transfected with GLucN-TIMP-1 and CD63-GLucC (TIMP-1/CD63), GLucN-HNF4 and HNF4-GLucC (HNF4 dimer), GLucN-TIMP-1 and HNF4-GLucC (T1/HNF4), GLucN-HNF4 and CD63-GLucC (HNF4/CD63), or without transfection (293FT NT). (**C**) Immunoblot analysis of TIMP-1 or CD63 using conditioned medium or cell lysates prepared from parental HEK293FT cells without transfection (293FT NT) or transfected with wild type TIMP-1 (T1), GLucN-TIMP-1 (G-T1), wild type CD63 (CD63) or CD63-GLucC (CD63-G). Transferrin receptor (TfR) was used as loading control. Lanes of the cell lysate analysis were separated as different exposures for CD63 (CD63) and CD63-GLucC (CD63-G) probes were used in order to clearly see the molecular weight ranges of CD63 that is heavily glycosylated and thus exhibits a diffuse distribution (see full immunoblot in Supplemental Fig. 2).

We initially identified CD63 as a TIMP-1 interacting protein by yeast two-hybrid (Y2H) screening^2^. For the domain mapping study, our current study utilized protein complementation assay (PCA) that allows protein synthesis, post-translational modifications and trafficking in mammalian cells, followed by a quantitative read-out of interactions between the proteins of interest. To measure interactions between TIMP-1 and CD63, we followed the basic design of luciferase bifurcation as optimized by I. Remy and S.W. Michnick^16^. Briefly, the 93 N-terminal amino acid residues of luciferase (GLucN) were fused to the N-terminus of TIMP-1 at Cys^1^ (GLucN-TIMP-1), while the C-terminal domain (aa 94–169) of luciferase (GLucC) was fused to the C-terminal region of CD63 truncated at Val^206^ (CD63-GLucC), as depicted in Fig. 1A as well as Supplemental Fig. 1. CD63-GLucC was designed not to include the C-terminal internalization motif and most of the 4^th^ TM domain of CD63^17^ so that it allows the GLucC fragment to be extracellular. Immunoblot analysis confirmed stable expression of these fusion-protein products in HEK293FT (Fig. 1C). Upon TIMP-1 interactions with CD63, bi-furcated luciferase fragments were re-combined and restored its activity (Fig. 1A,B). As a positive interaction control, hepatocyte nuclear factor 4α (HNF4), a transcription factor known to readily homodimerize, was used. Briefly, the GLucN (without a secretory signal peptide) and GLucC fragments were appended to the N-terminus and C-terminus of HNF4, respectively. As negative controls, cells were co-transfected with GLucN-TIMP-1 and HNF4-GLucC expression vectors (T1/HNF4), or GLucN-HNF4 and CD63-GLucC vectors (HNF4/CD63). Results shown in Fig. 1B demonstrated that there is no detectable recombined luciferase activity in the absence of specific interactions between proteins of interest (TIMP-1/CD63 or HNF4/HNF4).

**Figure 2:**
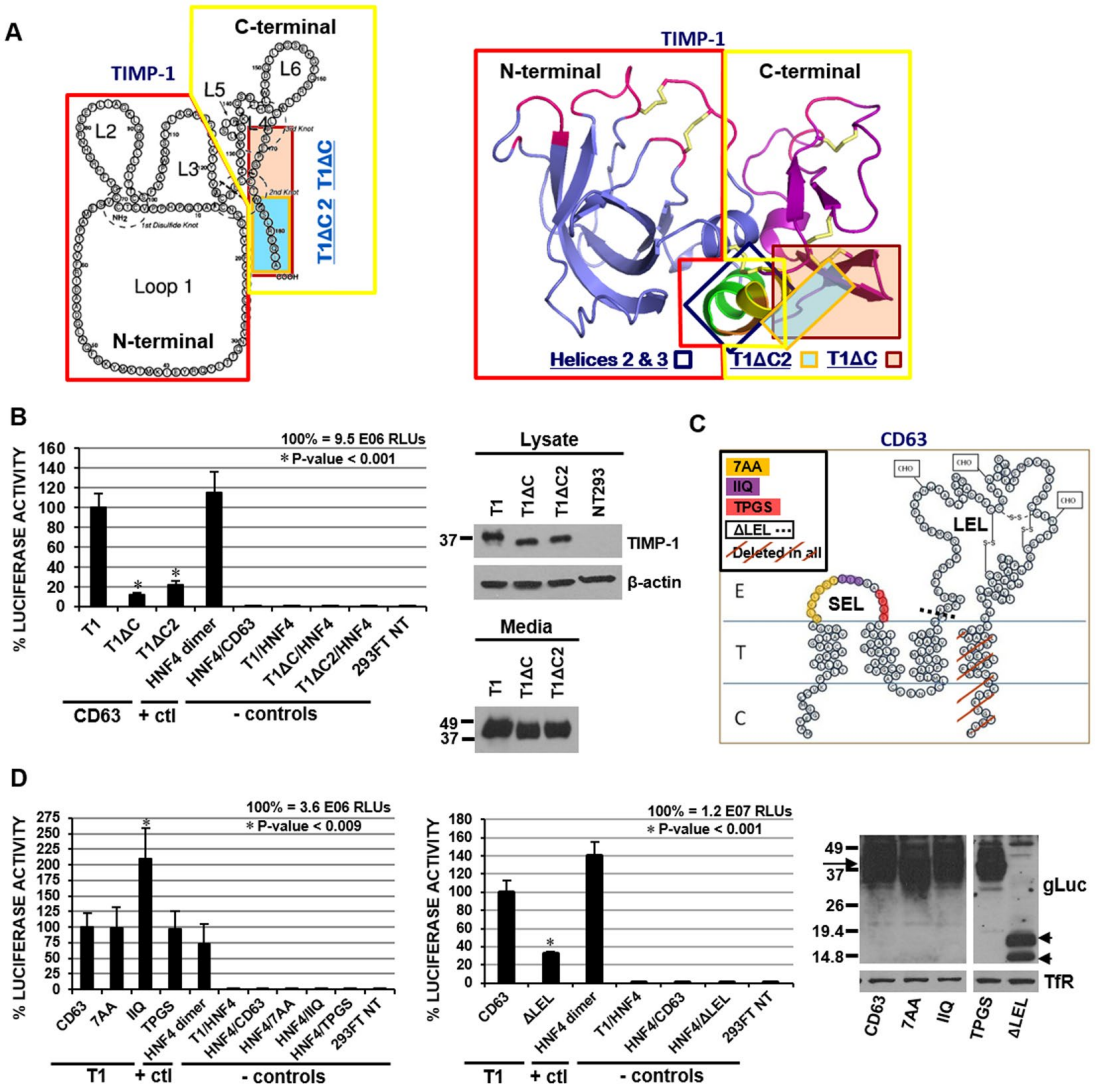
The C-terminus of TIMP-1 interacts with the extracellular large loop (LEL) of CD63. (**A**) 2-dimensional (left) and 3-dimensional (right) structure of TIMP-1, showing T1ΔC (17aa deletion, light orange) and T1ΔC2 (9aa deletion, light blue). 2D structural diagram of TIMP-1 adapted from Bodden *et al*.^21^. The N-terminal and C-terminal domains of TIMP-1 consist of loops 1–3 and 4–6, respectively. 3D structural diagram of TIMP-1 was made using PyMOL Molecular Graphics System based on the PDB entry 1UEA entered by Gomis-Ruth *et al*.^18^. The site occupied by MMP-3 catalytic domain in the crystal structure of MMP-3/TIMP-1 complex is shown in magenta in the N-terminal MMP inhibitory domain; helix 2 and helix 3 are shown in green and yellow respectively, and the connector between helices 2 and 3 is shown in orange; disulfide bridges are depicted in pale yellow. (**B**) PCA using cell lysates prepared from HEK293FT cells transfected with GLucN-TIMP-1 (T1), GLucN-T1ΔC (T1ΔC), or GLucN-T1ΔC2 (T1ΔC2) together with CD63-GLucC (CD63); GLucN-HNF4 and HNF4-GLucC (HNF4 dimer), GLucN-HNF4 and CD63-GLucC (HNF4/CD63), GLucN-TIMP-1 and HNF4-GLucC (T1/HNF4), GLucN-T1ΔC and HNF4-GLucC (T1ΔC/HNF4), GLucN-T1ΔC2 and HNF4-GLucC (T1ΔC2/HNF4), or without transfection (293FT NT). Immunoblot analysis of TIMP-1 using cell lysates or conditioned media from HEK293FT cells transfected with GLucN-TIMP-1 (T1), GLucN-T1ΔC (T1ΔC), GLucN-T1ΔC2 (T1ΔC2) or without transfection (293FT NT). (**C**) A diagram of CD63 depicting mutagenesis in the small extracellular loop (SEL) and the large extracellular loop (LEL) domains. Amino acid residues highlighted in color in the SEL were mutated to alanine residues and designated as 7AA, IIQ and TPGS accordingly. Separately, the entire LEL domain was removed for PCA domain analysis (ΔLEL). (**D)** PCA using cell lysates of HEK293FT cells transfected with luciferase fusion vectors as indicated (see Supplemental Fig. 1). Immunoblot analysis using anti-Gaussia Luciferase pAb in cell lysates of HEK293FT cells transfected with indicated wild type or mutant CD63-GLucC vectors (Right panel). Lanes are splitted because two separate immunoblots were used (see full immunoblot in Supplemental Fig. 2). Transferrin receptor (TfR) was used as loading control. For panels B and D, all values shown are the average of at least triplicate measurements. Error bars represent standard deviation. Significance was assessed as described in Materials and Methods with P < 0.05 being considered as significant.

The crystal structure of TIMP-1, complexed with the catalytic domain of MMP-3, revealed two distinct subdomains, the N-terminal wedge-shaped MMP binding domain and the C-terminal domain^18^. We previously demonstrated that the C-terminal domain of TIMP-1 (amino acids 126–184) is sufficient for its interaction with CD63^2^. Interestingly, however, the retention of the 2^nd^ and 3^rd^ helices (H2 & H3: L^110^-C^124^) of TIMP-1 is necessary for TIMP-1 activation of CD63-mediated intracellular signaling program^2,3^. Taken together, we hypothesized in this study that the C-terminus of TIMP-1 is the primary binding site for CD63 and the nearby helices H2 and H3 are required for its full engagement with the CD63 signaling complex on the cell surface (Fig. 2A). To address this hypothesis, we generated deletion mutants of TIMP-1 truncated after L^168^ (T1ΔC –deletion of 17 aa) or T^175^ (T1ΔC2 –deletion of 9aa) as depicted in Fig. 2A. Stable expression and secretion of these mutants were confirmed by immunoblot analysis using conditioned media (Fig. 2B). In comparison to full-length TIMP-1, these two mutants displayed drastic decreases in their ability to bind CD63 (T1ΔC and T1ΔC2 with loss of ~83% and ~79%, respectively). It is striking that T1ΔC and T1ΔC2, wherein all of TIMP-1’s cysteine bridges and its overall structure are expected to remain intact, drastically lost its CD63 binding activity (Fig. 2B). Thus, these results demonstrated that the 9 C-terminal amino acid residues are essential for TIMP-1 interactions with CD63.

Although the crystal structure of CD63 has not been successfully completed to this date, CD63 is thought to have 4 transmembrane domains and two extracellular loops as depicted in Fig. 2C. First, we examined whether the small extracellular loop (SEL, -Q^36^ LVLSQTIIQGATPGS^51^) of CD63 is critical for its interaction with extracellular TIMP-1 by alanine scanning mutagenesis. Amino acid residues in SEL were systematically substituted for alanine at Q^36^-T^42^, I^43^-Q^45^, and T^48^-S^51^ by site-directed mutagenesis and the corresponding substitution mutants were named 7AA, IIQ, and TPGS, respectively. Stable expression of those mutants was confirmed by immunoblot analysis (Fig. 2D). When we examined the ability of those SEL substitution mutants to interact with TIMP-1, none of them had significant decrease in its TIMP-1 binding activity; while it was noticed that substitution of IIQ to alanines increased its binding activity (Fig. 2D). This may be associated with a conformational change in CD63, making it more accessible to TIMP-1 binding.

Mutational analysis of the large extracellular loop (LEL) of CD63 is challenging due to its large size and the lack of knowledge about its 3-D structure. To examine the significance of the LEL for CD63’s ability to bind TIMP-1, our initial approach was to generate a deletion mutant devoid of LEL after the amino acid F^107^ (ΔLEL). As shown in Fig. 2D, CD63 in the absence of LEL significantly lost its ability to interact with TIMP-1. These results demonstrated that LEL, but not SEL, is a critical domain for CD63 interaction with TIMP-1. To narrow down the TIMP-1 interacting domain, the 6 cysteine residues within LEL of CD63 were mutated to serine, individually and in various combinations; two cysteine residues (C145,146 S; C169,170 S; C145,191 S; C146,170 S), three cysteine residues (C145,146,169 S; C146,169,170 S), four cysteine residues (C145,146,169,170 S), or five cysteine residues (C145,146,169,170,177 S). When C145 or C146 was mutated, especially when both C145 and C191 were mutated, CD63 significantly lost its ability to interact with TIMP-1. However, these changes were mostly due to variations in protein stability (data not shown). These results confirmed that the cysteine residues are important to maintain the integrity of the CD63 protein structure as predicted by its homology to other tetraspanins with conserved LEL cysteines^19,20^. However, very little information toward defining the TIMP-1 binding site within the LEL of CD63 was gained via mutagenesis of cysteine residues.

**Figure 3:**
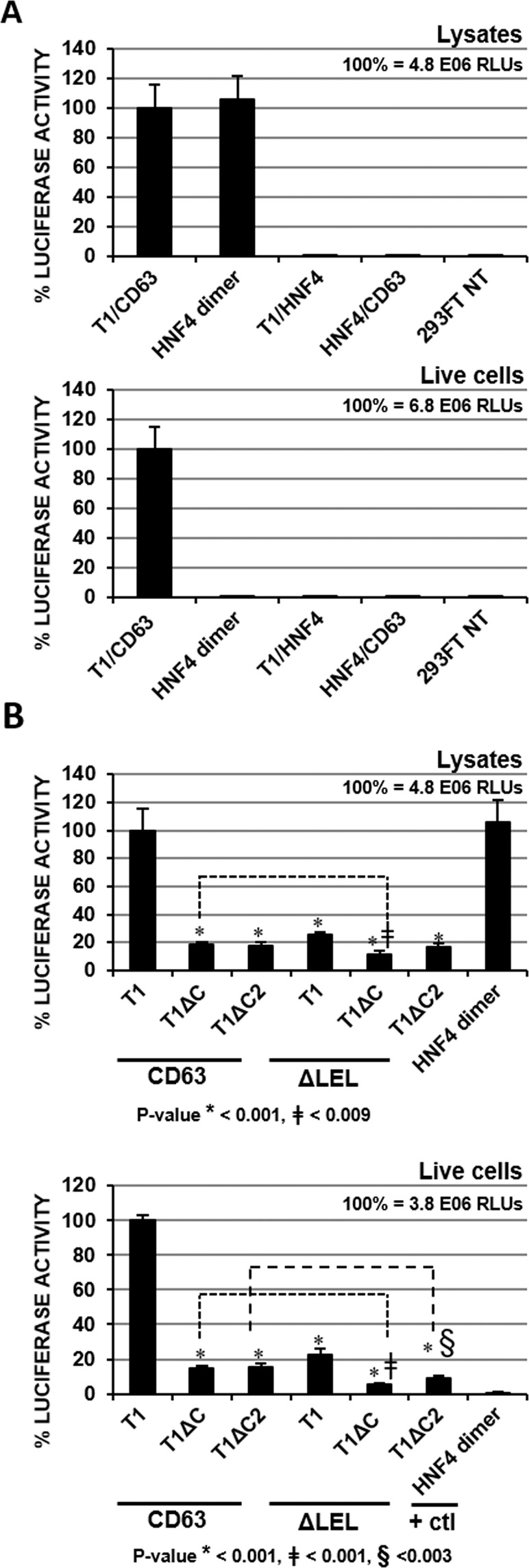
PCA measures TIMP-1 interactions with CD63 on the cell surface in TIMP-1’s C-terminus and CD63 LEL-dependent manners. (**A**) Luciferase activity was measured in cell lysates (top panel) or in live cells (bottom panel) of HEK293FT cells transfected with indicated luciferase fusion protein vectors or without transfection. (**B**) Luciferase activity was measured in cell lysates (top panel) or in live cells (bottom panel) of HEK293FT cells transfected with indicated luciferase fusion protein vectors. PCA for HNF4 dimerization was used as a positive control for interaction after cell compartment disruption (cell lysates) and as a negative control for extracellular biological interactions (live cells). Values shown are the average of at least triplicate measurements. Error bars represent standard deviation. Significance was assessed as described in Materials and Methods with P < 0.05 being considered as significant.

The above results by PCA using cell lysates show that the C-terminus of TIMP-1 and LEL of CD63 are critical for their interactions. It is of importance to demonstrate that interaction between GLucN-TIMP-1 and CD63-GLucC occurs on the cell surface as it does between endogenously expressed extracellular TIMP-1 and CD63 on the plasma membrane. When we performed PCA in live cells, HNF4 dimerization, expected to occur intracellularly, was not detected by live cell assay without cell membrane permeabilization. Importantly, TIMP-1/CD63 interaction was readily detected by a live cell assay in the C-terminus of TIMP-1- and LEL of CD63- dependent manners (Fig. 3), demonstrating their interactions on the cell surface.

**Figure 4:**
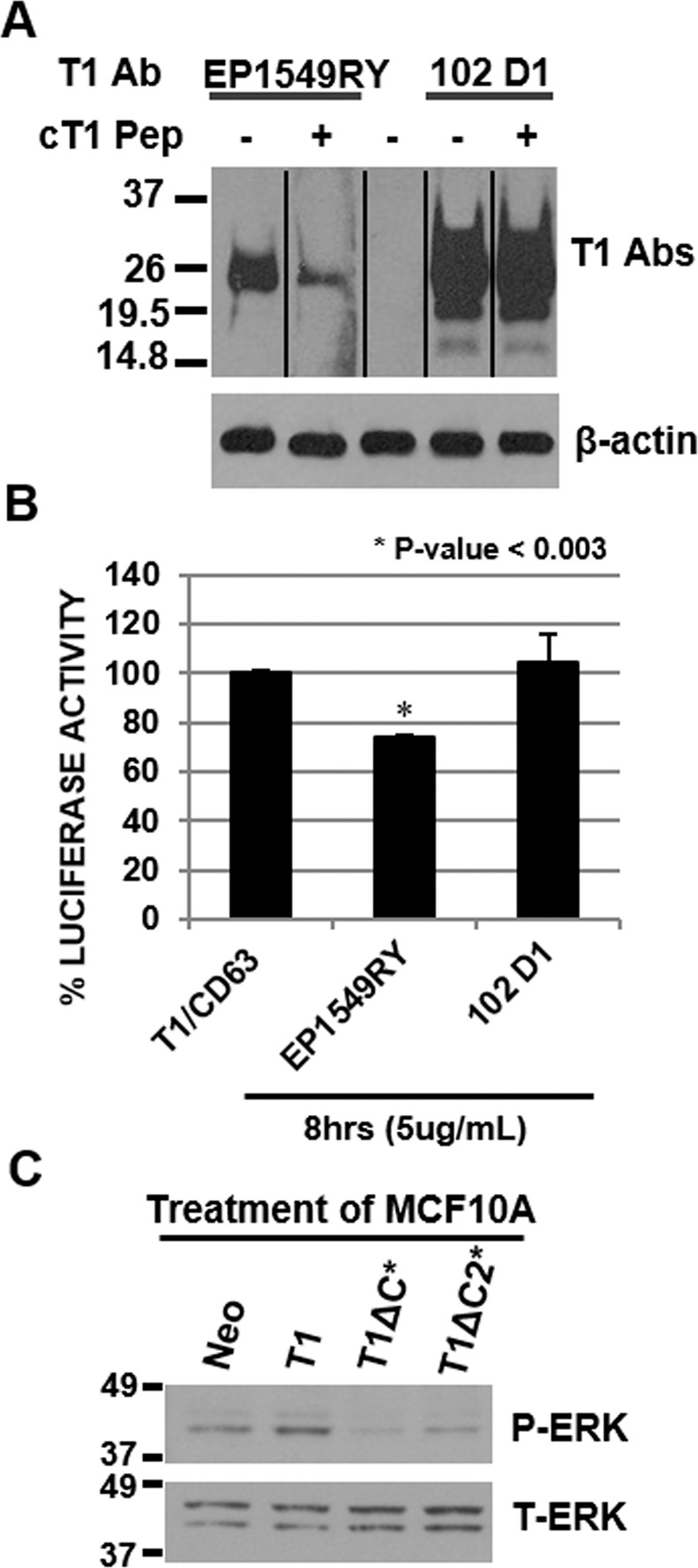
Antibody against the C-terminus of TIMP-1 interferes with TIMP-1’s interaction with CD63 and the C-terminus of TIMP-1 is essential for the activation of intracellular signaling. (**A**) Anti-TIMP-1 antibodies, EP1549RY and 102 D1, were pre-incubated with or without synthetic peptides corresponding to the 9 C-terminal amino acid residues of TIMP-1. Immunoblot analysis of TIMP-1 was performed using TIMP-1 overexpressing HEK293FT cell lysates. The nitrocellulose membrane was stained with Ponceau S and cut into strips. Each strip was probed with the indicated antibody. β-actin was used for loading control. (**B**) PCA for TIMP-1/CD63 interactions was performed in the presence or absence of C-terminal and non-C-terminal TIMP-1 Abs (EP1549RY and 102 D1, respectively). Values are shown after normalization to treatment with each antibody buffer alone and are representative of multiple experiments (with 100% at 1.3E07 and 1.6E07). Error bars represent standard deviation. (**C**) Immunoblot analysis of phopho- and total ERK using cell lysates of MCF10A cells treated with conditioned media collected from HEK293FT cells transfected with control vector (Neo), TIMP-1 (T1), T1ΔC, and T1ΔC2 expression vectors.

Next, we wished to evaluate the potential of targeting the C-terminus of TIMP-1 for inhibition of oncogenic signaling via CD63. First, we screened commercially available antibodies (Abs) for their ability to interact with the C-terminus of TIMP-1. When immunoblot analysis of TIMP-1 was performed using antibodies with or without pre-incubation with the synthetic peptide Acetyl-WQSLRSQIA-COOH representing the 9 C-terminal amino acids, anti-TIMP-1 Ab EP1549RY showed the most specific interactions with the C-terminus of TIMP-1 among Abs tested in this study (Fig. 4A). Next, we examined whether anti-TIMP-1 Ab EP1549RY can interfere with TIMP-1 binding to CD63. As shown in Fig. 4B, PCA analysis in live cells demonstrated that EP1549RY, but not 102 D1 whose epitope is not the C-terminus of TIMP-1, interfered with TIMP-1 for its binding to CD63 on the cell surface. It should be noted that although this reduction was reproducible and statistically significant, it was not robust. We surmise that the inefficient inhibition is the result of lower than the desirable concentration of EP1549RY antibody.

Lastly, we examined the significance of TIMP-1’s C-terminus for the induction of intracellular signaling. Human breast epithelial cell line MCF10A was treated with conditioned media prepared from HEK293FT cells transfected with wild type TIMP-1, T1ΔC or T1ΔC2 expression vector. HEK293FT cells were chosen for transfection since they do not express endogenous TIMP-1 proteins at a detectable level. As previously reported^6^, wild-type TIMP-1 readily activated ERK in MCF10A cells, whereas TIMP-1 lacking its C-terminal 17aa (T1ΔC) or 9aa (T1ΔC2) failed to activate ERK (Fig. 4C), supporting the notion that the C-terminus of TIMP-1 is critical for its binding to and activation of CD63-mediated signaling.

We envision that our findings may lead to the development of mechanism-based therapeutic intervention that specifically targets TIMP-1’s oncogenic functions while preserving its tumor-suppressive MMP inhibitory activity. In light of the emerging clinical evidence that TIMP-1 is highly expressed and serves as a predictive marker of poor prognosis in patients with many types of cancers including lymphoma, melanoma, lung, colon, pancreatic, breast and prostate cancers, our finding may have broad implications in human cancers.

## Supplementary information

Supplemental Material.

Supplemental Figure Legends.

## Data Availability

The data generated within the current study are available from the corresponding author upon request.
