# Supplementary material for: Establishment of Structure-Function Relationship of Tissue Inhibitor of Metalloproteinase-1 for Its Interaction with CD63: Implication for Cancer Therapy
Source: Sci Rep. 2020 Feb 7;10:2099. doi: 10.1038/s41598-020-58964-x (PMC7005868; doi:10.1038/s41598-020-58964-x)
Supplement: Supplementary file 1 — Supplemental Material. [file 41598_2020_58964_MOESM1_ESM.pdf]

Establishment of Structure-Function Relationship of Tissue Inhibitor of Metalloproteinase-1 for Its Interaction with CD63: Implication for Cancer Therapy

Richard B. Warner[1,2,3], Abdo J. Najy[1], Young Suk Jung[1,4] , Rafael Fridman[1], Seongho Kim[2], and

Hyeong-Reh Choi Kim*[1]

Department of Pathology[1] and Oncology[2] Barbara Ann Karmanos Cancer Institute, Wayne State University School of Medicine, Detroit, MI 48201.

Warner et al.
Supplemental Figure 1

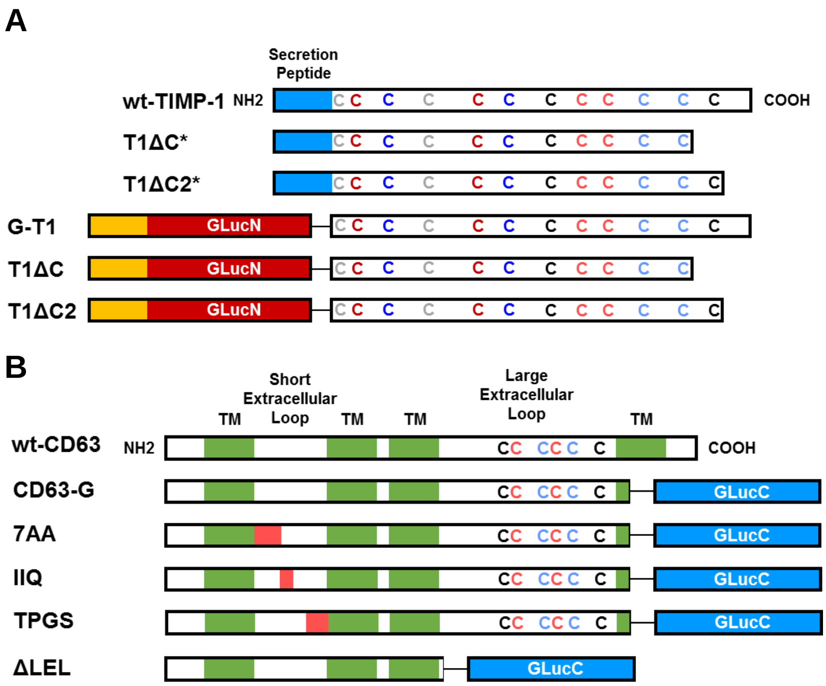

Warner et al.
Supplemental Figure 2

A. From Fig 1

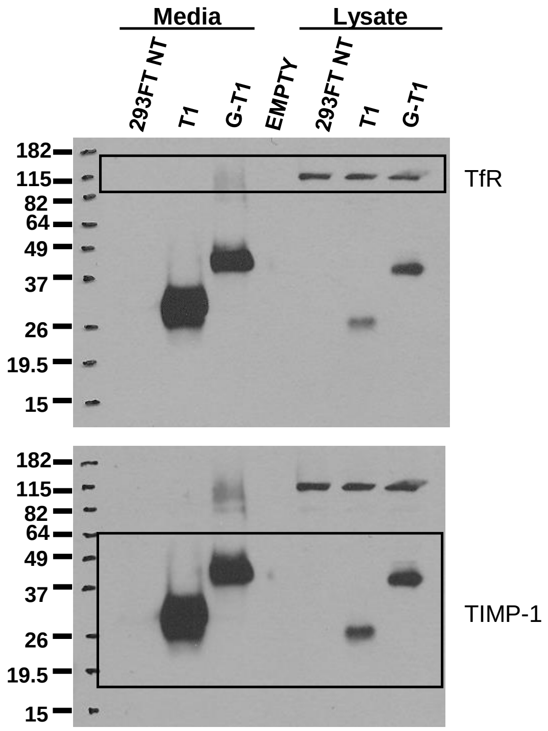

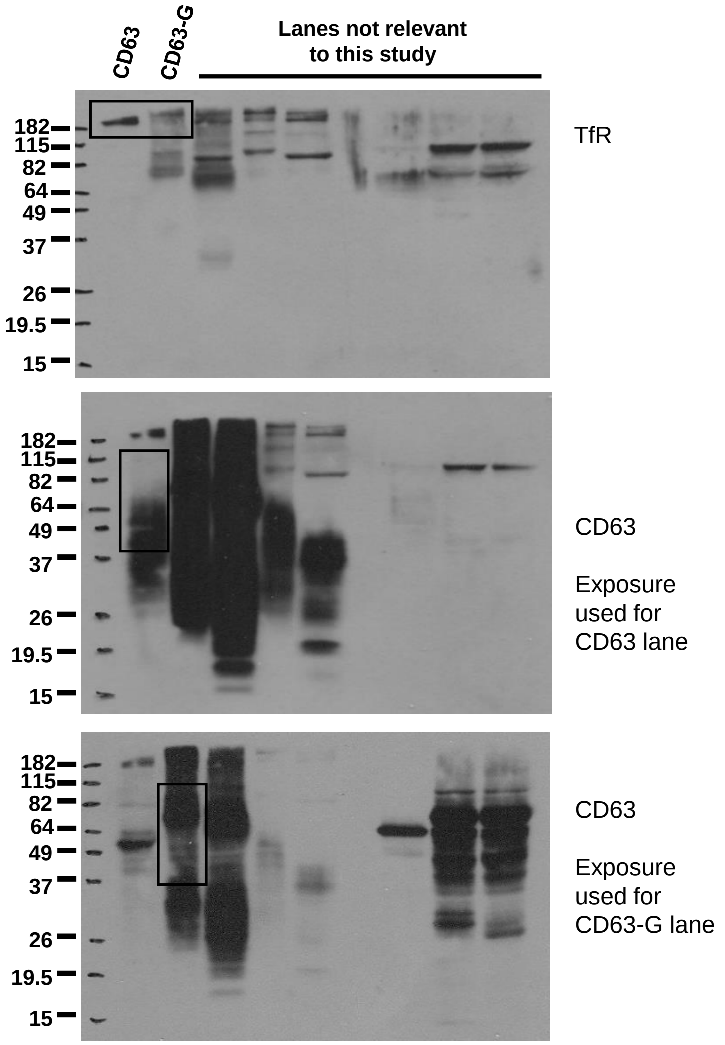

B. From Fig 2B

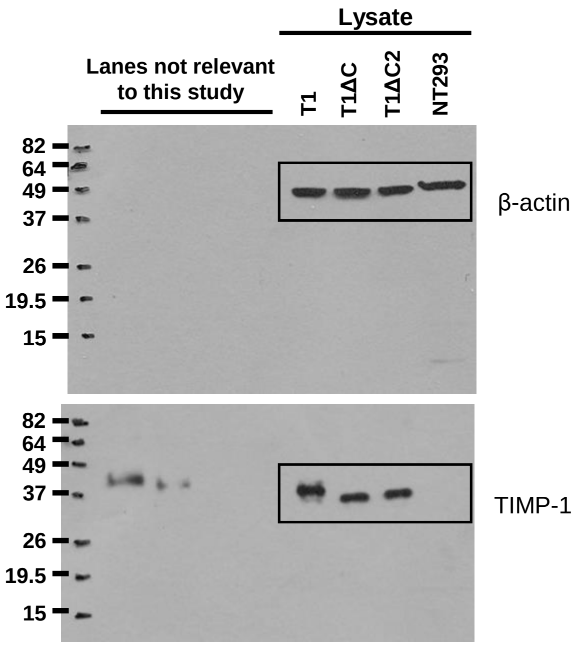

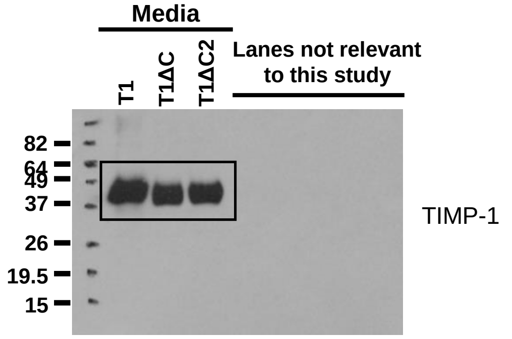

Warner et al.
Supplemental Figure 2

C. From Fig 2D

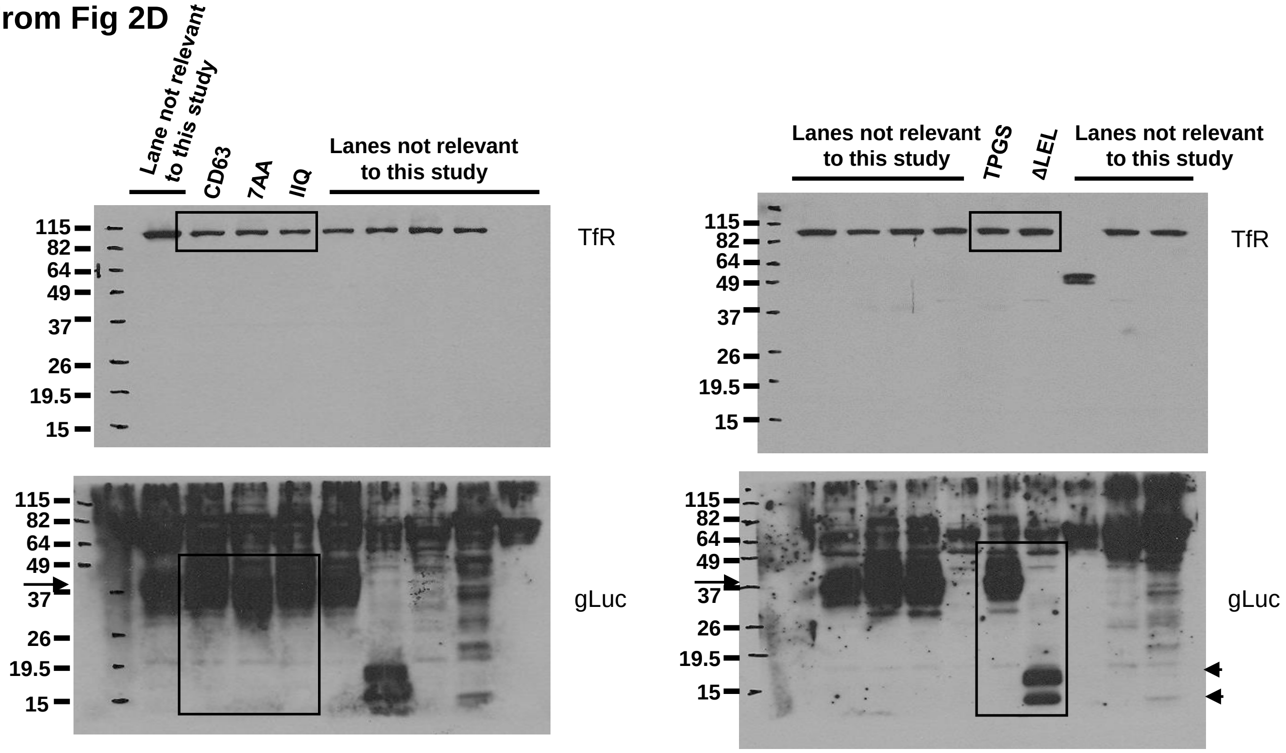

D. From Fig 4A

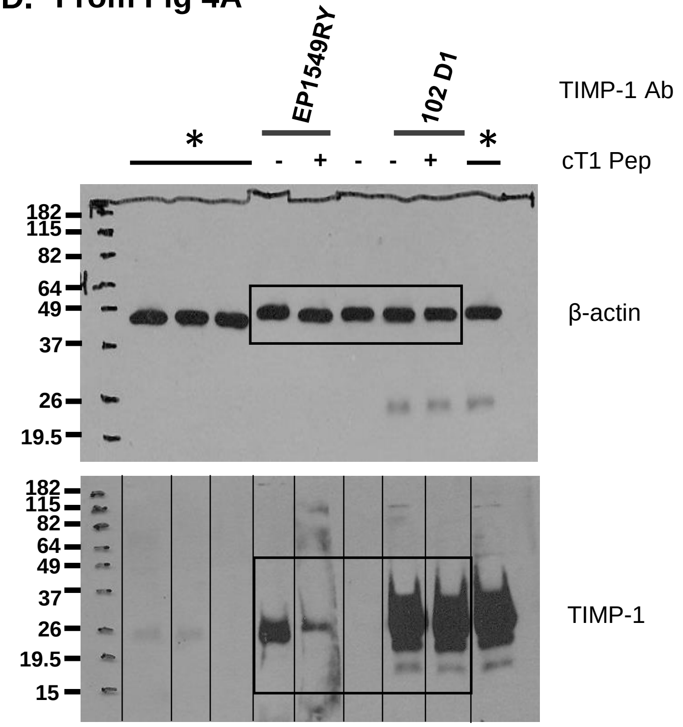

*Lanes not relevant to the study

E. From Fig 4C

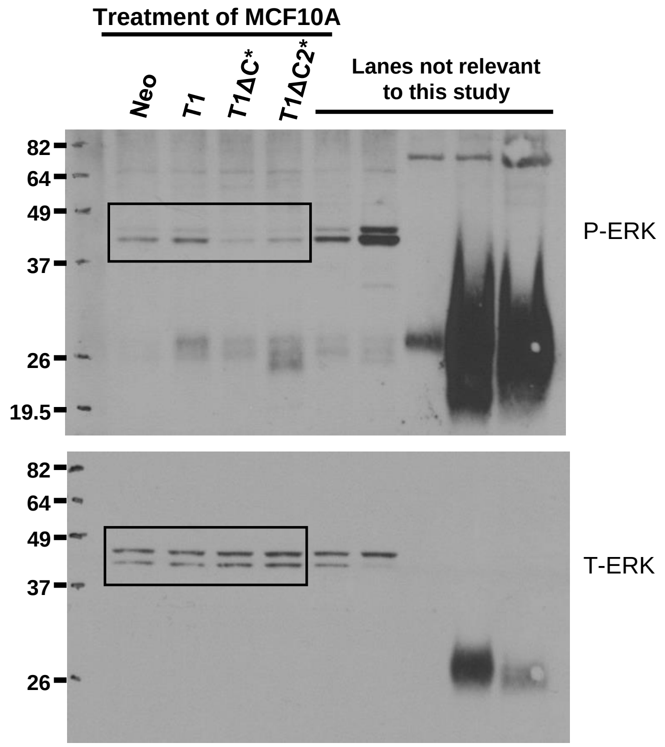

**SUPPLEMENTAL TABLE 1**		
CD63 MUTATION PRIMERS		
7AA	F	5'-GGG ACT GAT TGC CGT GGG TGT CGG GGC AGC GGC TGC CGC GGC TGC GGC CAT AAT CCA GGG GGC TAC CCC-3'
7AA	R	5'-GGG GTA GCC CCC TGG ATT ATG GCC GCA GCC GCG GCA GCC GCT GCC CCG ACA CCC ACG GCA ATC AGT CCC-3'
IIQ	F	5'-CAG CTT GTC CTG AGT CAG ACC GCA GCC GCG GGG GCT ACC CCT GGC TCT CTG TTG-3'
IIQ	R	5'-CAA CAG AGA GCC AGG GGT AGC CCC CGC GGC TGC GGT CTG ACT CAG GAC AAG CTG-3'
TPGS	F	5'-ATA ATC CAG GGG GCT GCC GCT GCC GCT CTG TTG CCA GTG G-3'
TPGS	R	5'-CCA CTG GCA ACA GAG CGG CAG CGG CAG CCC CCT GGA TTA T-3'
PCA SUB-CLONING AND TRUNCATION PRIMERS		
CD63-HINDIII	F	5'-GAA AGC TTG CCA TGG CGG TGG AA-3'
CD63-AGEI	R	5'-GAA CCG GTT CTG AGC CTC CTC CGC CTG AAC CTC CTC CTC CTA CCA CCA GCA CAT T-3'
CD63-ΔLEL	R	5'-GAA CCG GTT CTG AGC CTC CTC CGC CTG AAC CTC CTC CTC CAA ACA CAT AGC CAG C-3'
AGEI-KOZAK-GLUC-SIGNALPEPTIDE	F	5'-CGC CCA CCG GTC ACC ATG GGA GTC AAA GTT CTG TTT GCC-3'
TIMP-1-HINDIII	F	5'-GAA AGC TTG CAC CTG TGT CCC A-3'
T1ΔC	R	5'-GAT CTA GAC AGG CAG GCA AGG TG-3'
T1ΔC2	R	5'-GAT CTA GAG GTG CAC AGC CCT GG-3'
FOR TIMP-1 DELETION IN EXPRESSION VECTOR		
T1ΔC-W/STOP	R	5'-GAT CTA GAC TAC AGG CAG GCA AGG TG-3'
T1ΔC2-W/STOP	R	5'-GAT CTA GAC TAG GTG CAC AGC CCT GG-3'
