# Supplementary material for: Establishment of Structure-Function Relationship of Tissue Inhibitor of Metalloproteinase-1 for Its Interaction with CD63: Implication for Cancer Therapy
Source: Sci Rep. 2020 Feb 7;10:2099. doi: 10.1038/s41598-020-58964-x (PMC7005868; doi:10.1038/s41598-020-58964-x)
Supplement: Supplementary file 2 — Supplemental Figure Legends. [file 41598_2020_58964_MOESM2_ESM.docx]

**Establishment of Structure-Function Relationship of Tissue Inhibitor of Metalloproteinase-1 for Its Interaction with CD63: Implication for Cancer Therapy**

Richard B. Warner^1,2,3^, Abdo J. Najy^1^, Young Suk Jung^1,4^ , Rafael Fridman^1^, Seongho Kim^2^, and Hyeong-Reh Choi Kim*^1^

Department of Pathology^1^ and Oncology^2^ Barbara Ann Karmanos Cancer Institute, Wayne State University School of Medicine, Detroit, MI 48201.

**Supplemental Figure 1. Diagrams of protein-fragment complementation assay vectors.**

(**A**) Vector construction for the N-terminal fragment of humanized Gaussia Luciferase (GLucN) and TIMP-1 fusion proteins. Shown in colors are: 23aa secretion peptide of TIMP-1 (blue) and the N-terminal fragment of humanized Gaussia Luciferase (GLucN) (red) with a humanized secretion peptide (light orange). GLucN was fused to the first cysteine (C^1^) of TIMP-1 [wild type or with deletion of the 17 (T1ΔC*) or 9 (T1ΔC2*) C-terminal amino acid residues] via a flexible linker of 10aa (GGGGSGGGGS). “C” represents a cysteine residue, and the color-matched “C”s depict the di-sulfide linkage. (**B**) Vector construction for the CD63 and C-terminal fragment of humanized Gaussia Luciferase (GLucC) fusion proteins. Shown in colors are: four transmembrane (TM) domains (green) and mutated regions (7AA, IIQ, TPGS) in the short extracellular loop (red). The C-terminal fragment of humanized Gaussia Luciferase (GLucC, blue) was fused after V^206^ of CD63 via the same flexible linker used above. For ΔLEL, the fusion of GLucC was made after residue F^107^. “C” represents a cysteine residue, and the color-matched “C”s depict the di-sulfide linkage.

**Supplemental Figure 2. Full-length immunoblots for the analyses in Figures 1, 2, and 4.**

Panels displayed in the main manuscript figures are indicated by a rectangle. **A)** Immunoblot analysis of TIMP-1, CD63 and transferrin receptor (TfR) presented in Figure 1C. Lower exposure (left, top panel) and higher exposure (left, bottom panel) of the blot were used for TfR and TIMP-1 panels, respectively. The right, top panel was used for TfR. Higher exposure (right, middle panel) and lower exposure (right, bottom panel) of the blot were used for CD63 and CD63-G lanes, respectively. **B)** Immunoblot analysis of TIMP-1 and β-actin presented in Figure 2B. Cell lysates were immunoblotted with TIMP-1 (left, bottom panel) then re-probed with β-actin (left, top panel) for loading control. TIMP-1 expression in the media of transfected cells was analyzed using immnoblotting for TIMP-1. **C)** Immunoblot analysis of Gaussia Luciferase (gLuc) and transferrin receptor (TfR) presented in Figure 2D. Cell lysates of wild type CD63, 7AA or IIQ mutants were immunoblotted with gLuc (left, bottom panel) then re-probed with TfR (left, top panel) for loading control. Cell lysates of the TPGS or ΔLEL CD63 mutants were immunoblotted with gLuc (right, bottom panel) then re-probed with TfR (right, top panel) for loading control. **D)** Immunoblot analysis of TIMP-1 and β-actin presented in Figure 4A. Cell lysates of were immunoblotted with TIMP-1 (left, bottom panel) or with β-actin (left, top panel) for loading control. **E)** Immunoblot analysis of P-ERK (right, top panel) and T-ERK (right, bottom panel) presented in Figure 4C.
